# Persistent Food Insecurity and Overweight/Obesity Risk in Offspring of Individuals With Gestational Diabetes

**DOI:** 10.1111/ijpo.70138

**Published:** 2026-07-26

**Authors:** Keally Haushalter, Jaimie N. Davis, Yeyi Zhu, Joan C. Lo, Erin A. Hudson, Alexis King, Louise Greenspan, Jami Josefson, Erica P. Gunderson

**Affiliations:** ^1^ Department of Nutritional Sciences University of Texas at Austin Austin Texas USA; ^2^ Division of Research Kaiser Permanente Northern California Pleasanton California USA; ^3^ Kaiser Permanente Bernard J. Tyson School of Medicine Pasadena California USA; ^4^ The Kaiser Permanente Medical Group, Kaiser Permanente Medical Center San Francisco California USA; ^5^ Lurie Children's Hospital of Chicago, Northwestern University Feinberg School of Medicine Chicago Illinois USA

**Keywords:** “food insecurity”, “gestational diabetes”, “longitudinal”, “obesity”

## Abstract

**Objective:**

Assess how changes in food insecurity in the first 2 years of life are associated with overweight and obesity among children 6–11 years old born to mothers with gestational diabetes mellitus (GDM).

**Methods:**

This secondary analysis utilised data from the Study of Women, Infant Feeding and Type 2 Diabetes Mellitus after GDM pregnancy, a prospective cohort of 1033 mother–child dyads. Food insecurity was assessed 6–9 weeks, 1 year and 2 years postpartum. Children were categorized as having persistent food security (food secure all visits), persistent food insecurity (food insecure all visits), or transient food insecurity (experienced both food security and insecurity). Overweight, obesity and severe obesity were classified using height and weight at 6–11 years old. Linear and logistic regressions estimated the relationship between food insecurity groups and BMI percentile, overweight, obesity and severe obesity.

**Results:**

Among 748 youth, 72% had persistent food security, 18% had transient food insecurity and 10% had persistent food insecurity. Compared to those with persistent food security, those with persistent food insecurity had higher odds of overweight (aOR = 2.81, [1.36, 5.80]), obesity (aOR = 2.17, [1.05, 4.47]) and severe obesity (aOR = 2.62, [1.02, 6.74]) (*p* < 0.05).

**Conclusions:**

Screening and addressing food insecurity during early childhood may help identify children at risk for overweight, obesity and severe obesity among children exposed to GDM.

**Trial Registration:** ClinicalTrials.gov identifier: NCT019967030

**Abbreviations**
BMI: body mass index
CDC: Centers for Disease Control
EHR: electronic health records
GDM: gestational diabetes mellitus
KPNC: Kaiser Permanente Northern California
OGTT: Oral glucose tolerance test
SWIFT: The Study of Women, Infant Feeding and Type 2 Diabetes after Gestational Diabetes Pregnancy
USDA: US Department of Agriculture
WIC: The Special Supplemental Nutrition Program for Women, Infants and Children

## 1 Introduction

Food insecurity, defined by the US Department of Agriculture (USDA) as a ‘household‐level economic and social condition of limited or uncertain access to adequate food’, affected over 13.5% (18 million) of US households in 2023 and is more prevalent among households with children (17.9%, approximately 6.5 million households) [1, 2]. The high prevalence of food insecurity is alarming, as it has been associated with negative economic and health outcomes. In 2016, the Centers for Disease Control (CDC) estimated that food insecurity led to an extra $52.9 billion in excess health care expenditures [3]. In addition, a study of almost 30 000 US children (2–17 years old) found that children with food insecurity, compared to those with food security, had worse general health, more emergency department visits, delayed medical care and a higher prevalence of chronic health conditions [4]. Further, recent studies in children and adults have found food insecurity status to be associated with a higher risk of obesity [5, 6, 7].

While research has shown food insecurity to increase one's risk for obesity, most of the research is cross‐sectional, meaning it only assesses one's food insecurity status at one time [8, 9]. This is a limitation as food insecurity status changes. Among food‐insecure households, about one‐fourth experience chronic food insecurity, while the other three‐fourths face food insecurity for an average of 7 months out of the year [10]. Further, a study that followed over 500 preschoolers throughout an academic year found that 54% of the households had persistent food security, 21% had persistent food insecurity and 25% had changes in their food insecurity status (transient food insecurity) [11]. These studies highlight how food insecurity status is dynamic and changes within a single household. Since measuring food insecurity once fails to capture how most households experience food insecurity, evaluating how transient and persistent food insecurity are associated with obesity may provide a more accurate relationship between these measures.

Few studies have examined the longitudinal relationship between food insecurity status and obesity parameters in children, and those that have reported conflicting results. One study followed ~5000 socioeconomically disadvantaged children from birth to 22 years old and found that those with persistent food security had a lower body mass index (BMI) compared to those with transient or persistent food insecurity [12]. A 2022 systematic review examined the longitudinal relationship between food insecurity and obesity among US children, 1–19 years old, and found mixed results, concluding that the relationship may be dose‐dependent, with a longer exposure to food insecurity being associated with higher odds for obesity [8].

The elevated risk of obesity for children who experience food insecurity may be exacerbated by other factors. For example, we previously found that exposure to gestational diabetes mellitus (GDM), a condition in which mothers have elevated glucose levels during pregnancy, independently increases the risk of overweight and obesity during childhood [13]. In addition, mothers who are food insecure during pregnancy have a higher risk of developing GDM [14]. While both food insecurity and exposure to GDM in utero are independently associated with an increased risk for overweight and obesity, it is unclear if experiencing food insecurity after exposure to GDM further exacerbates this risk. Given that food insecurity is already associated with an increased risk for overweight and obesity, and exposure to GDM in utero is also associated with a higher risk for overweight and obesity, experiencing both conditions may come with an elevated risk. To fill this gap, this study aimed to evaluate how persistent and transient food insecurity, compared to persistent food security, over the first two years of life, was associated with the prevalence of overweight, obesity and severe obesity in 6–11‐year‐old youth who were exposed to GDM.

## 2 Materials and Methods

This secondary analysis utilised data from The Study of Women, Infant Feeding and Type 2 Diabetes after Gestational Diabetes Pregnancy (SWIFT). Full details regarding recruitment, inclusion criteria, study procedures, visits and assessments are published elsewhere [15, 16]. In short, the SWIFT study evaluated the associations for lactation intensity and duration with progression to type 2 diabetes in women after a GDM pregnancy and identified modifiable risk factors to prevent future progression to type 2 diabetes. At study baseline, 1033 pregnant women diagnosed with GDM from 13 Kaiser Permanente Northern California (KPNC) medical centres were recruited and enrolled in SWIFT between July 2008 and December 2011. Women aged 20–45 at delivery (2008–2011), diagnosed with GDM by Carpenter and Coustan criteria [17], who delivered a singleton, live birth ≥ 35 weeks' gestation and met other criteria were eligible to participate in SWIFT [15, 16]. Participants provided written informed consent and completed three in‐person research visits at 6–9 weeks, 1 year and 2 years postpartum as part of the SWIFT study. Research visits included a 2‐h 75 g research oral glucose tolerance test (OGTT), standardised anthropometric assessments, and validated surveys at each research visit, and, in the first year, monthly mailed surveys on infant feeding and maternal and child health. KPNC electronic health records (EHR) were also utilized to obtain additional clinical health data during the pregnancy course and delivery, and longer‐term follow‐up in women and their children. The SWIFT study was approved by the Institutional Review Board at Kaiser Permanente Northern California and registered as a clinical trial (NCT019967030). The measurements relevant to this secondary analysis are described below.

### 2.1 Children's Height and Weight

Children's height and weight were measured at their KPNC well‐child pediatric outpatient examinations or at the youth's SWIFT research visit between 6 and 11 years old. The most recent exam (closest to 11 years old) was used if the child had multiple exams. BMI percentile was calculated using age and sex specific guidelines from the CDC [18]. Based on these guidelines, children were categorized as having normal weight (< 85th percentile), overweight (≥ 85th percentile and < 95th percentile) or obesity (≥ 95th percentile). To further characterise the severity of obesity, in accordance with the CDC definition and guidelines, severe obesity was defined as a child's BMI above the ≥ 120% of the CDC 95th percentile [19, 20].

### 2.2 Food Insecurity

Food insecurity was assessed by surveys at research visits at ages 6–9 weeks, 1 year and 2 years using the USDA Food Sufficiency Question which asks the mothers ‘Within the past 12 months, which one of these statements best describes the food eaten by your family?’ with answer options of: ‘We have enough food to eat and the kinds of food we want’, ‘We have enough food to eat, but NOT always the kinds of food we want to eat’, ‘Sometimes we don't have enough food to eat’, ‘Often we don't have enough food to eat’ and ‘Prefer not to answer’ [21]. The USDA Food Sufficiency Question has been used in multiple national studies, including the Nationwide Food Consumption Survey and the Food Security Supplement to the Census Bureau's Current Population Survey, to estimate food insecurity and is correlated with other measures of food insecurity and diet quality [22, 23, 24].

Mothers reported their household income through the question ‘What was your total combined income for ALL family members in your household for the past 12 months from all sources, before taxes?’ and selected which income bracket they fell into (ranging from < 5000 to > 150 000). Mothers also reported how many individuals lived in their homes by writing a numeric answer to the question, ‘How many persons currently live in your home, including yourself?’ Using both the household size and their income, the participant's poverty‐to‐income ratio was calculated using the respective poverty guideline from the year the survey was completed, by dividing their income by the poverty level for their respective household size [21].

In line with USDA guidelines, participants were classified as food secure if they answered, ‘We have enough food to eat and the kinds of food we want’ and had an income‐to‐poverty ratio greater than two. If participants answered, ‘We have enough food to eat, but NOT always the kinds of food we want to eat’, ‘Sometimes we don't have enough food to eat’, or ‘Often we don't have enough food to eat’, they were classified as food insecure regardless of income‐to‐poverty ratio. If participants selected ‘We have enough food to eat and the kinds of food we want’, but had an income‐to‐poverty ratio equal to or less than two, they were classified as food insecure.

This analysis included mother–child dyads who completed at least two out of the three food security questionnaires. If they were classified as food secure on all completed surveys, they were labelled as having ‘persistent food security’. Similarly, if they were classified as food insecure on all completed surveys, they were labelled as having ‘persistent food insecurity’. If their food insecurity status switched on any of the surveys (being a mix of food secure and food insecure), they were classified as having ‘transient food insecurity’.

### 2.3 Mothers Measurements

At each in‐person research visit (6–9 weeks, 1 year and 2 years postpartum), trained research assistants followed standardised protocols to assess maternal anthropometry and body composition and administered surveys to participants. The mothers' pre‐pregnancy BMI and perinatal outcomes were obtained from their EHR. Maternal educational attainment, maternal nativity and insurance status were also assessed through surveys completed at 6–9 weeks postpartum [15].

### 2.4 Lactation Intensity Ratio

Given that exclusive breastfeeding is less common among those with food insecurity [25], and that breastfeeding is associated with a decreased risk for childhood obesity [26], breastfeeding intensity was important to consider in this analysis. Breastfeeding intensity was assessed through mailed monthly surveys from birth to 12 months, which asked mothers to report the frequency of breastfeeding and formula feeding (the number of bottles of breastmilk and formula supplementation) per 24 h for the past 7 days. Each month, participants received a score ranging from zero to one that reflected the fractional portion of milk feeds that were breastmilk, with zero indicating that 0% of the milk feeds were from breastmilk, and one indicating that 100% of the milk feeds were from breastmilk [27]. Cumulative breastfeeding intensity from birth through 12 months was calculated by summing participants' scores each month, resulting in a cumulative score that ranged from zero to 12, with zero indicating that they never breastfed and 12 indicating that they exclusively breastfed from birth to 12 months.

### 2.5 WIC Participation

Participants' participation in The Special Supplemental Nutrition Program for Women, Infants and Children (WIC) was measured at 6–9 weeks, 1 year and 2 years postpartum in a written survey. Participants were asked, ‘Do you receive benefits from the WIC program?’ with answer options of ‘no’, ‘yes’ and ‘no answer’. If participants selected ‘yes’ at any of the three visits, they were categorized as a ‘WIC recipient’.

### 2.6 Insurance Status

Participants' insurance status was accessed from the mother's EHR at baseline. Participants were categorised as having insurance from either purchasing it themselves or through their employer, versus through MediCal (California's Medicaid program) or the military.

### 2.7 Covariates

Covariates were selected a priori based on what influences food insecurity, overweight and obesity and GDM, and which variables that showed significant differences between food insecurity groups at *p* < 0.20. The covariates selected for this analysis included mother's pre‐pregnancy BMI (continuous), child's ethnicity/race (White, Asian, Black, Hispanic, Multiracial/Other), mother's educational attainment (less than a college degree, a college degree or more), mother's nativity (inside the US, outside the US, unknown), whether they received WIC (WIC recipient, never received), insurance status (self/employer vs. MediCal/Military) and lactation intensity ratio (sum of birth‐12 months).

### 2.8 Statistical Analysis

Descriptive statistics (ANOVA and chi‐square test) were used to determine sociodemographic differences between those included in the analytic sample versus those enrolled in the study but excluded from the analytic sample. To assess sociodemographic differences between the three food insecurity groups, descriptive statistics (ANOVA and chi‐square test) were used. For groups with significant differences, Bonferroni correction (categorical variables) and Tukey HSD (continuous variables) tests were used to determine which groups significantly differed.

For continuous outcomes (BMI percentile), multivariable linear regression assessed the relationship between food insecurity status and BMI percentile. For categorical outcomes (overweight, obesity and severe obesity), one multinomial logistic regression was used to assess the association of food insecurity groups with the odds of having overweight, obesity or severe obesity, compared to normal weight. For the linear and the multinomial logistic regressions, unadjusted and adjusted odds ratios were estimated, adjusting for the covariates described above.

For each model, collinearity was assessed by ensuring the variance inflation factor was < 5. In addition, all observations were independent, and for the multinomial logistic regression, all categorical outcomes were mutually exclusive. To ensure there were no sparse cell issues, all outcomes were checked to have a cell size > 5. Finally, the models used covariate data that was collected at baseline, so there was no missing data in the analytic sample.

## 3 Results

### 3.1 Analytic Sample

Of the 1033 mother–child dyads who were enrolled in the study, 748 youth had attended a KPNC well‐child visit between 6 and 11 years old, where height and weight were measured, and had completed at least two food insecurity screeners in the first 2 years postpartum. Compared to those enrolled in the study but not included in the analytic sample (*n* = 285), those in the analytic sample (*n* = 748) were more likely to have a college degree or more (40% vs. 52%, *p* < 0.001) and had a higher lactation intensity ratio (4.8 vs. 6.1, *p* < 0.001). There were no other sociodemographic differences between those included and excluded from the analytic sample (data not shown).

Table 1 describes the sociodemographic and BMI markers associated with the 748 youth included in this analysis. On average, the youth were 9.6 (±1.4) years of age, and 53.3% were male. Asian (36%) and Hispanic (34%) were the most common racial/ethnic groups, followed by White (20%), Black (9%) and Multiracial/Other (1%). At birth, most of the youth were appropriate size for gestational age (76.9%), 14.4% were large for gestational age and 8.7% were small for gestational age. Youth had an average age‐ and sex‐specific BMI percentile of 68.6 (±1.3). Fifty‐six percent of the youth had normal BMI, 18% had overweight, 26% had obesity and 7% had severe obesity. Mothers had an average pre‐pregnancy BMI of 29.6 kg/m^2^ (±7.3) and had similar percentages across gestational weight gain recommendations for pre‐pregnancy BMI (31% gained less than recommended, 34% were within recommendations, and 34% gained more than recommended). On average, mothers received their diagnosis of GDM at week 25.3 (±7.1) of their pregnancy. Most mothers (93%) had private insurance, and close to 30% of mothers received WIC. Approximately half (52%) had a college degree or more; 51% were born inside the US, and the average lactation intensity ratio score for 12 months was 6.09 (±4.6) out of a total range of zero to 12.

**TABLE 1:** Sociodemographic characteristics of analytic sample and food insecurity groups.

	Analytic sample	Persistent food security	Transient food insecurity	Persistent food insecurity	*p* ^a^
	*N* = 748	*N* = 539	*N* = 133	*N* = 76	
Mother's characteristics	Mean (±SD)				
Pre‐pregnancy BMI	29.64 (7.31)	28.85 (6.89)	31.42 (7.83)	32.15 (8.20)	**< 0.001**
Week of GDM diagnosis	25.30 (7.05)	25.32 (6.79)	25.65 (7.36)	24.50 (8.21)	0.520
	*N* (%)				
Pregnancy weight change					0.416
< Recommendation	234 (31.28%)	160 (29.68%)	47 (35.34%)	27 (35.53%)	
Within recommendation	257 (34.36%)	196 (36.36%)	39 (29.32%)	22 (28.95%)	
> Recommendation	257 (34.36%)	183 (33.95%)	47 (35.34%)	27 (35.53%)	
WIC					**< 0.001**
Never a WIC recipient	517 (69.12%)	430 (79.78%)	64 (48.12%)	23 (30.26%)	
Received WIC at any point	231 (30.88%)	109 (20.22%)	69 (51.88%)	53 (69.74%)	
Insurance status					**0.037**
Self/employer	699 (93.45%)	511 (94.81%)	121 (90.98%)	67 (88.16%)	
MediCal/Military	49 (6.55%)	28 (5.19%)	12 (9.02%)	9 (11.84%)	
Education attainment					**< 0.001**
Less than college	357 (47.73%)	203 (37.66%)	92 (69.17%)	62 (81.58%)	
College degree or more	391 (52.27%)	336 (62.34%)	41 (30.83%)	14 (18.42%)	
Nativity					**< 0.001**
Born outside the US	362 (48.40%)	258 (47.87%)	62 (46.62%)	42 (55.26%)	
Born inside the US	378 (50.53%)	280 (51.95%)	69 (51.88%)	29 (38.16%)	
Unknown	8 (1.07%)	1 (0.19%)	2 (1.50%)	5 (6.58%)	
Child's characteristics	*N* (%)				
Sex					0.503
Male	393 (52.54%)	287 (53.25%)	64 (48.12%)	42 (55.26%)	
Female	355 (47.46%)	252 (46.75%)	69 (51.88%)	34 (44.74%)	
Race/ethnicity					**< 0.001**
White	146 (19.52%)	115 (21.34%)	21 (15.79%)	10 (13.16%)	
Asian	269 (35.96%)	229 (42.49%)	30 (22.56%)	10 (13.16%)	
Black	68 (9.09%)	42 (7.79%)	17 (12.78%)	9 (11.84%)	
Hispanic	256 (34.22%)	148 (27.46%)	61 (45.86%)	47 (61.84%)	
Multiracial/other	9 (1.20%)	5 (0.93%)	4 (3.01%)	0 (0.00%)	
Size for gestational age					0.608
Appropriate	575 (76.87%)	414 (76.81%)	100 (75.19%)	61 (80.26%)	
Large	108 (14.44%)	74 (13.73%)	23 (17.29%)	11 (14.47%)	
Small	65 (8.69%)	51 (9.46%)	10 (7.52%)	4 (5.26%)	
Overweight (≥ 85th–< 95th)	132 (17.65%)	87 (16.14%)	24 (18.05%)	21 (27.63%)	0.057
Obesity (≥ 95th)	197 (26.34%)	126 (23.38%)	40 (30.08%)	31 (40.79%)	**0.003**
Severe Obesity (≥ 120%)	55 (7.35%)	31 (5.75%)	13 (9.77%)	11 (14.47%)	**0.012**
	Mean (±SD)				
Child age (years)	9.61 (1.38)	9.62 (1.37)	9.53 (1.48)	9.63 (1.32)	0.759
Lactation intensity ratio	6.09 (4.55)	6.45 (4.54)	5.14 (4.31)	5.19 (4.73)	**0.002**
BMI percentile (%)	68.59 (29.68)	65.92 (30.20)	71.87 (28.75)	81.73 (23.14)	**< 0.001**

*Note:* WIC stands for The Special Supplemental Nutrition Program for Women, Infants, and Children. Child's size for gestational age was defined using the 2017 World Health Organization guidelines. BMI categorisation is based on Center for Disease Control age‐and‐sex specific guidelines for a child's BMI percentile. Severe obesity is defined as a BMI percentile ≥ 120% of the 95th percentile. Lactation intensity ratio is the monthly score summed from birth to 12 months.

^a^ ANOVA and chi‐square tests were used to assess group difference across continuous and categorical variables, respectively.

### 3.2 Differences Between Food Insecurity Groups

Among 748 infants, 539 (72.1%) were classified as having persistent food security, 133 (17.8%) as having transient food insecurity and 76 (10.2%) as having persistent food insecurity from birth through 2 years. The significant sociodemographic differences between the food insecurity groups are displayed in Table 2. Compared to those with persistent food security, those with persistent food insecurity had a higher prevalence of obesity (23.4% vs. 40.8%, *p* = 0.005) and severe obesity (5.8% vs. 14.5%, *p* = 0.03) at age 6–11 years. In addition, youths' BMI percentile was also significantly higher among those with persistent food insecurity than those with persistent food security (65.9 vs. 81.7, *p* < 001). Compared to youth with transient food insecurity, youth with persistent food insecurity had a higher BMI percentile at ages 6–11 years (71.9 vs. 81.7, *p* = 0.05).

**TABLE 2:** Significant sociodemographic characteristics between food insecurity groups.

	Analytic sample	Persistent food security (PFS)	Transient food insecurity (TSI)	Persistent food insecurity (PFI)	*p* ^a^	Pairwise comparisons^b^
	*N* = 748	*N* = 539	*N* = 133	*N* = 76		
Mother's characteristics	Mean (±SD)					
Pre‐pregnancy BMI	29.64 (7.31)	28.85 (6.89)	31.42 (7.83)	32.15 (8.20)	**< 0.001**	**PFS vs. TFI: < 0.001** **PFS vs. PFI: < 0.001**
WIC		*N* (%)			**< 0.001**	**PFS vs. TFI: < 0.001**
Never a WIC recipient	517 (69.12%)	430 (79.78%)	64 (48.12%)	23 (30.26%)		**PFS vs. PFI: < 0.001**
Received WIC at any point	231 (30.88%)	109 (20.22%)	69 (51.88%)	53 (69.74%)		
Insurance status					**0.037**	NS
Self/employer	699 (93.45%)	511 (94.81%)	121 (90.98%)	67 (88.16%)		
MediCal/Military	49 (6.55%)	28 (5.19%)	12 (9.02%)	9 (11.84%)		
Education attainment					**< 0.001**	**PFS vs. TFI: < 0.001**
Less than college	357 (47.73%)	203 (37.66%)	92 (69.17%)	62 (81.58%)		**PFS vs. PFI: < 0.001**
College degree or more	391 (52.27%)	336 (62.34%)	41 (30.83%)	14 (18.42%)		
Nativity					**< 0.001**	**PFS vs. PFI: < 0.001**
Born outside the US	362 (48.40%)	258 (47.87%)	62 (46.62%)	42 (55.26%)		
Born inside the US	378 (50.53%)	280 (51.95%)	69 (51.88%)	29 (38.16%)		
Unknown	8 (1.07%)	1 (0.19%)	2 (1.50%)	5 (6.58%)		
Child's characteristics	*N* (%)					
Race/ethnicity					**< 0.001**	**PFS vs. TFI: < 0.001**
White	146 (19.52%)	115 (21.34%)	21 (15.79%)	10 (13.16%)		**PFS vs. PFI: < 0.001**
Asian	269 (35.96%)	229 (42.49%)	30 (22.56%)	10 (13.16%)		
Black	68 (9.09%)	42 (7.79%)	17 (12.78%)	9 (11.84%)		
Hispanic	256 (34.22%)	148 (27.46%)	61 (45.86%)	47 (61.84%)		
Multiracial/other	9 (1.20%)	5 (0.93%)	4 (3.01%)	0 (0.00%)		
Obesity (≥ 95th)	197 (26.34%)	126 (23.38%)	40 (30.08%)	31 (40.79%)	**0.003**	**PFS vs. PFI: 0.005**
Severe obesity (≥ 120%)	55 (7.35%)	31 (5.75%)	13 (9.77%)	11 (14.47%)	**0.012**	**PFS vs. PFI: 0.03**
	Mean (±SD)					
Lactation intensity ratio	6.09 (4.55)	6.45 (4.54)	5.14 (4.31)	5.19 (4.73)	**0.002**	**PFS vs. TFI: 0.01**
BMI percentile (%)	68.59 (29.68)	65.92 (30.20)	71.87 (28.75)	81.73 (23.14)	**< 0.001**	**PFS vs. PFI: < 0.001** **TFI vs. PFI: 0.05**

*Note:* Child's size for gestational age was defined using the 2017 World Health Organization guidelines. BMI categorisation based on Center for Disease Control age‐and‐sex specific guidelines for a child's BMI percentile. Obesity is defined as a BMI percentile ≥ 95. Severe obesity defined as a BMI percentile ≥ 120% of the 95th percentile. Lactation intensity ratio is the monthly score summed from birth to 12 months.

Abbreviations: GDM, gestational diabetes mellitus; PFI, persistent food insecurity; PFS, persistent food security; TFI, transient food insecurity; WIC, The Special Supplemental Nutrition Program for Women, Infants and Children.

^a^ ANOVA and chi‐square tests were used to assess group difference across continuous and categorical variables, respectively.

^b^ Bonferroni correction was used for categorical variables, Tukey HSD correction for continuous variables.

Compared to those who had persistent food security, those with transient food insecurity and persistent food insecurity had significantly higher rates of mothers receiving WIC (secure: 20.2%, transient: 51.9%, insecure: 69.7%, *p* < 0.001), and mothers who had a college degree or more (secure: 62.3%, transient: 30.8%, insecure: 18.4%, *p* < 0.001). In addition, mothers of youth with transient and persistent food insecurity had significantly higher pre‐pregnancy BMI than mothers of youth with persistent food security (secure: 28.9, transient: 31.4, insecure: 32.2, *p* < 0.001). Youths' race/ethnicity was also significantly different between those who had persistent food security and those who had either transient or persistent food insecurity (*p* < 0.001). Further, compared to those with persistent food security, those with persistent food insecurity had a lower prevalence of mothers born inside the US (52.0% vs. 38.2%, *p* < 0.001). Finally, those with transient food insecurity had a significantly lower lactation intensity ratio than those with persistent food security (5.2 vs. 6.45, *p* = 0.01).

### 3.3 Food Insecurity Predicting Markers of Obesity

**FIGURE 1:**
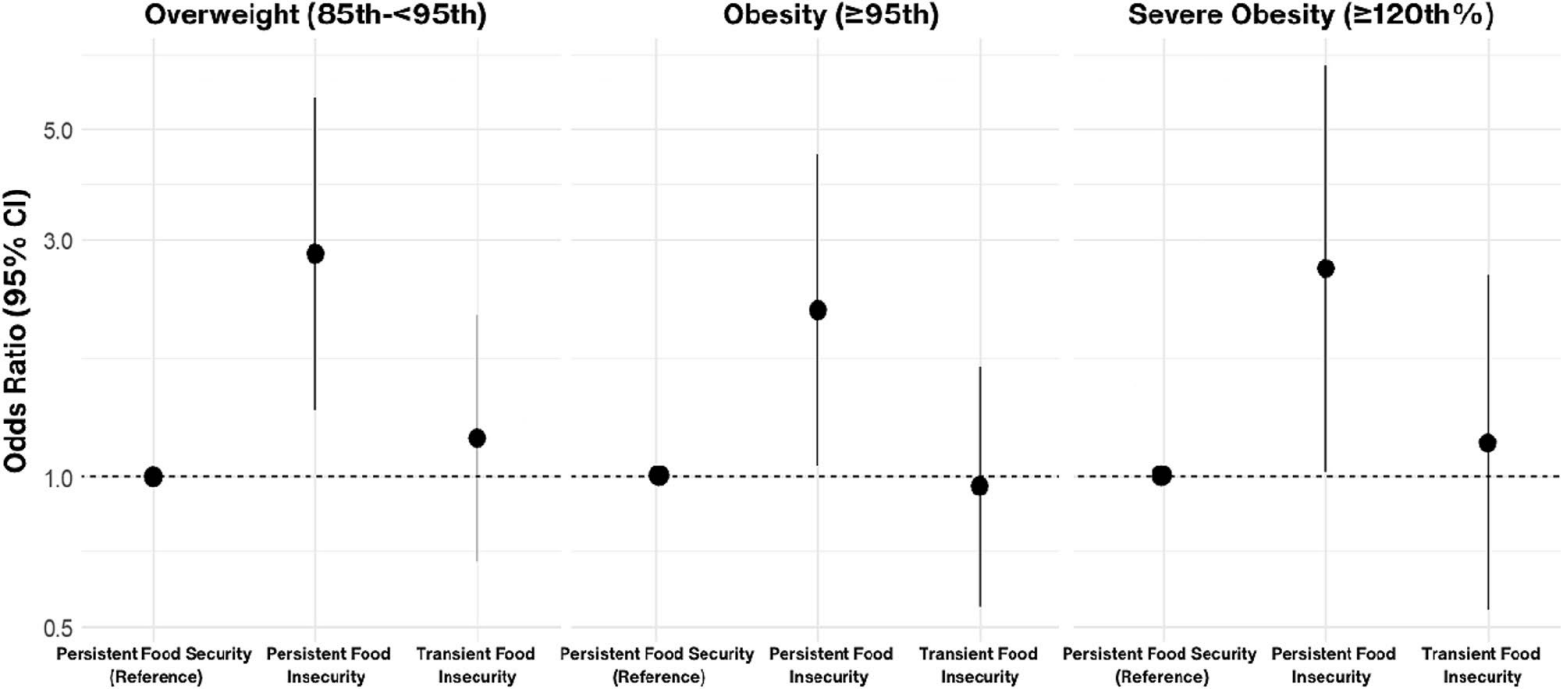
Relationship between food insecurity and the odds of having overweight, obesity, or severe obesity. Persistent Food Security is the reference group. Overweight is defined as BMI percentile ≥ 85th and < 95th, obesity is defined as BMI percentile ≥ 95th, and severe obesity is defined as BMI percentile ≥ 120% of the 95th percentile. 539 were classified as having persistent food security, 133 as having transient food insecurity and 76 as having persistent food insecurity. Analysis adjusted for mothers' pre‐pregnancy BMI, child's ethnicity/race, mother's education attainment, mother's nativity, insurance status, whether they received WIC, and lactation intensity ratio sum.

The unadjusted and adjusted linear and logistic regressions of food insecurity groups' associations with youth BMI percentile are presented in Table 3 and Figure 1. After controlling for covariates, those with persistent food insecurity had significantly higher odds of overweight (aOR = 2.81, 95% CI [1.36, 5.80], *p* = 0.005), obesity (aOR = 2.17, 95% CI [1.05, 4.47], *p* = 0.04) and severe obesity (aOR = 2.62, 95% CI [1.02, 6.74], *p* = 0.04) than those with persistent food security. There was no significant difference in odds of overweight, obesity, or severe obesity between those with persistent food security and transient food security in the adjusted models. Compared to those with persistent food security, there was no significant difference in BMI percentile between those with persistent food insecurity or transient food insecurity (*p* > 0.05).

**TABLE 3:** Food insecurity group associations with BMI percentile, overweight, obesity and severe obesity at 6–11 years old.

Variable	BMI percentile		Overweight^a^		Obesity^b^		Severe obesity^c^	
	B (95% CI)^d^	*p* ^e^	aOR (95% CI)^f^	*p* ^e^	aOR (95% CI)^f^	*p* ^e^	aOR (95% CI)^f^	*p* ^e^
Food insecurity group								
Persistent food security	1.00	Referent	1.00	—	1.00	—	1.00	—
Transient food insecurity	0.61 (−4.95, 6.18)	0.83	1.20 (0.68, 2.12)	0.54	0.96 (0.55, 1.67)	0.88	1.17 (0.54, 2.54)	0.69
Persistent food insecurity	6.95 (−0.36, 14.26)	0.06	2.81 (1.36, 5.80)	**0.005**	2.17 (1.05, 4.47)	**0.04**	2.62 (1.02, 6.74)	**0.04**

^a^ Defined as BMI percentile ≥ 85th and < 95th (*n* = 132).

^b^ Defined as BMI percentile ≥ 95th (*n* = 197).

^c^ Defined as BMI percentile ≥ 120% of the 95th percentile (*n* = 55).

^d^ Multivariate linear regression.

^e^ *p* < 0.05 are significant, denoted with bolded numbers.

^f^ Multinomial logistic regression (normal vs. overweight vs. obesity vs. severe obesity); All models control for mother's pre‐pregnancy BMI, child's race/ethnicity (White, Asian, Black, Hispanic, Multiracial/Other), mother's education attainment (less than a college degree, college degree or more), mother's nativity (outside the US, inside the US, unknown), participation in the Special Supplemental Nutrition Program for Women, Infants and Children (WIC) status (did not receive WIC, received WIC), insurance status (self/employer vs. MediCal/Military), and lactation intensity ratio (monthly score summed from birth to 12 months).

## 4 Discussion

This study of 748 primarily racial and ethnic minority youth analysed how changes in food insecurity during the first 2 years of life were associated with the prevalence of overweight, obesity and severe obesity in youth ages 6–11 years old who were exposed to GDM. Compared to those with persistent food security, those with persistent food insecurity during early childhood had significantly higher odds of being classified as having overweight, obesity, or severe obesity at 6–11 years old. There was no significant difference in BMI percentile or the risk of overweight, obesity, or severe obesity between those with persistent food security and transient food insecurity. These findings highlight how early exposures to food insecurity among youth exposed to GDM in utero are related to weight status, and therefore health risks, later in life.

This study aligns with current literature suggesting that the relationship between food insecurity and obesity among children may be dose‐dependent [8]. However, this finding conflicts with a study that found that children with transient food insecurity, but not persistent food insecurity, have a higher risk of obesity than children with persistent food security [12]. Therefore, more research is needed to understand if the amount of time one is food insecure or if changes in food insecurity are more predictive of one's health.

Among US youth aged 2–19 years old, 16.1% are overweight, 19.3% have obesity and 6.1% have severe obesity [28]. The increased prevalence of overweight, obesity and severe obesity in this already high‐risk population of children exposed to GDM, as evidenced by this study and others [29], is concerning, as it increases their risk for developing other health conditions, such as type 2 diabetes, asthma, sleep apnea, joint problems and obesity later in childhood, adolescence and adulthood [30]. In addition, children with overweight and obesity also have an increased risk for adverse mental health conditions, such as anxiety, depression and low self‐esteem [30]. These compounding physical and mental health conditions not only increase the risk of a low quality of life, but also increase their medical care costs, making it harder to afford other cost‐of‐living expenses, such as food and housing [31, 32, 33, 34].

The increased risk for overweight, obesity and severe obesity among persistently food‐insecure children may stem from their diet. A cross‐sectional study of over 4500 2–19‐year‐olds found that food‐insecure children, compared to food‐secure children, consume more refined grains and less fruits, vegetables and protein [35]. Another cross‐sectional study of ~3000 fourth and fifth‐grade students found that food‐insecure children ate, on average, 118 cal, 4 g of fat and 8 g of sugar more than their food‐secure counterparts each day [36]. Therefore, food insecurity is likely to coexist with a poor diet, which may increase one's risk for overweight and obesity.

The poor dietary intake of food‐insecure children may result from the barriers these families face. First, compared to food‐secure families, food‐insecure families are more likely to purchase food at convenience stores and small markets, which have lower availability of healthy, fresh items, such as fruits and vegetables [37, 38]. Second, in a focus group of nine food‐insecure parents, not knowing how to properly prepare healthy meals and not having time to cook healthy meals were reported as barriers to a healthy diet [39]. Finally, as the cost of food has risen overall, the cost of healthy foods has risen more than the cost of unhealthy foods [40, 41]. Therefore, food‐insecure families may only be able to afford the cheapest items, which are often calorie‐dense, nutrient‐poor, highly processed foods [41, 42].

To improve the diet quality and health outcomes associated with food insecurity, various food assistance programmes, such as the Supplemental Nutrition Assistance Program (SNAP) and food pantries, provide low‐income families with vouchers to purchase food or food directly. While these programmes are successful in reducing food insecurity, they have not reduced dietary and health disparities [43]. Food assistance program users, compared to income‐eligible non‐users, have higher rates of chronic diseases and nutritionally poor diets [44, 45, 46, 47]. This may be because food assistance programmes, such as food banks and food pantries, do not have the proper equipment or storage to consistently offer fresh produce and therefore must rely on shelf‐stable items, which are often calorie‐dense, high in fat, sodium and added sugars and low in fibre, vitamins and minerals [47, 48, 49, 50, 51, 52]. Further, food assistance program users also may lack the time, transportation, kitchen equipment and nutrition or cooking knowledge to obtain and prepare healthy meals [53, 54]. Therefore, future studies are needed to understand how to efficiently and effectively provide food‐insecure families with healthy options that are affordable, obtainable, easy to prepare and desirable. It is also important to note that this study evaluated participation by mothers in the WIC program during the first 2 years postpartum, which suggests WIC did not alleviate food insecurity for this vulnerable population. Given this finding, it is important to analyse how WIC, a nutrition program designed for mothers and children, can be better tailored and more accessible to alleviate food insecurity and reduce health disparities.

In addition to providing families with access to a healthy diet, the consistency of this access is important. Food‐insecure families go through a monthly cycle where they have access to ample food for the first 3 weeks and have limited food for the last week [55, 56]. In anticipation of times when food is limited, individuals over‐consume when food is available, a consumption pattern known as famine‐feast that has been associated with an increased risk for obesity [57, 58]. Therefore, ensuring families have consistent access to healthy foods is essential to prevent the development of the famine‐feast eating cycle.

This study also found that persistent food insecurity in the first 2 years of life was related to an increased risk of overweight and obesity almost a decade later, but not transient food insecurity. Many studies analysing food insecurity and health outcomes are cross‐sectional, only taking one's concurrent food insecurity status into account [8, 9]. However, this finding highlights the importance of repeatedly screening for food insecurity early in life, due to its impact on the child's health later in life. Further, these findings suggest that the amount of time one is food insecure may relate to their risk for adverse health conditions. Therefore, consistently measuring food insecurity to determine whether it is periodic or chronic is essential to understand the impact it may have on their health. This is especially critical for groups who are more likely to have adverse health outcomes, such as those exposed to GDM. Youth exposed to GDM experience an intrauterine environment with elevated glucose concentrations. As a result, the foetus may develop hyperinsulinemia, which promotes adipogenesis, fat deposition and accelerated growth [59, 60]. Further, developing hyperinsulinemia in utero may induce long‐term metabolic programming through alterations in insulin sensitivity, appetite regulation, energy metabolism and epigenetic pathways, all of which place the youth at a higher risk for developing overweight and obesity, which could be further exacerbated by food insecurity [61, 62].

### 4.1 Strengths and Limitations

The strength of this study stems from its large sample of primarily racial and ethnic minority youth who were exposed to GDM. In addition, the longitudinal nature of this study is unique as it analyses changes in food insecurity. Given that the majority of food‐insecure households experience food insecurity periodically, not chronically, analysing changes in food insecurity may provide a more accurate understanding of how exposure to food insecurity impacts youth's health. In addition, the study analysed health status both continuously (BMI percentile) and categorically (overweight, obesity, severe obesity). This is a strength, as while food insecurity was associated with the risk of overweight, obesity and severe obesity, it was not associated with BMI percentile. This inconsistency reflects that BMI percentiles are bounded and compressed at the upper end of the distribution, limiting the ability to distinguish variation among children with high BMI percentiles. Categorical classifications, on the other hand, capture clinically meaningful thresholds and therefore may be more sensitive to identifying differences associated with food insecurity. As a result, the observed association may indicate that food insecurity is related to an increased likelihood of crossing obesity‐related thresholds, rather than a uniform increase in BMI percentile.

However, this study is not without its limitations. The study measured food insecurity only during the first 2 years of the child's life. Therefore, the youth's food insecurity status could have changed afterwards, but it was not reflected in the study. Second, this study only included children exposed to GDM. While this means the findings may not apply to youth who were not exposed to GDM, offspring of mothers with GDM have a higher risk for adverse cardiometabolic conditions, which heightens the clinical impact of this research. Also, this secondary analysis assessed markers of obesity, but did not analyse other markers of youth's health, such as glucose control, plasma lipids, or blood pressure. Given that both children with food insecurity and children exposed to GDM have a higher risk for other adverse cardiometabolic conditions, future studies should analyse how changes in food insecurity are associated with changes in other markers of health to provide a more holistic understanding of the effects of food insecurity. We also did not analyse the children's dietary intake or physical activity at older ages, which could be a possible mechanism between food insecurity and their risk for overweight and obesity.

Further, those in the analytic sample, compared to those enrolled in the study but excluded, had a significantly higher prevalence of having a college degree or more and a significantly higher lactation intensity ratio. While these differences may suggest possible attrition bias to a slightly more educated and healthier group, there were no differences among any other sociodemographic variable, suggesting the analytic sample may still be representative of those enrolled in the study. Finally, approximately one‐third of the sample was Asian and the majority (52%) had a college degree, which are groups that traditionally have lower rates of food insecurity and obesity [63, 64, 65]. However, this is still representative of the Northern California population, and even among this sample, a significant relationship was found between food insecurity and higher odds of overweight, obesity and severe obesity.

## 5 Conclusion

Overall, this study found that experiencing persistent food insecurity, compared to persistent food security, during the first 2 years of life was associated with an increased risk of overweight, obesity and severe obesity at 6–11 years old in youth exposed to GDM. This finding supports the need to screen for food insecurity consistently in early life and intervene with programmes that alleviate food insecurity. Policies should focus on improving the access and availability of healthy foods to families with food insecurity during this crucial time. Future interventions are needed to identify effective strategies to help children who face food insecurity, particularly those exposed to GDM, mitigate adverse health outcomes.

## Funding

The analyses were supported by grants from the National Institute of Diabetes, Digestive and Kidney Diseases, R01DK122700, R21DK103171 and R01DK11840, the National Institute of Child Health and Human Development, R01HD050625 (to E.P.G.) and the ADA, all awarded to E.P.G.

## Disclosure

The authors have nothing to report.

## Conflicts of Interest

The authors declare no conflicts of interest.

## Supporting information


**Data S1:** CONSORT 2025 checklist item description.

## Data Availability

The data that support the findings of this study are available on request from the corresponding author. The data are not publicly available due to privacy or ethical restrictions.
